# An audit into the management of diabetes mellitus at Broadmoor Hospital

**DOI:** 10.1192/bjo.2021.223

**Published:** 2021-06-18

**Authors:** Elliott Carthy, Callum Ross

**Affiliations:** 1 Oxford Health NHS Foundation Trust; 2 Broadmoor Hospital, Crowthorne Berks

## Abstract

**Aims:**

Diabetes mellitus confers a twofold excess risk of cardiovascular disease - the leading cause of premature mortality in those with severe mental illness. Inpatients in forensic settings often have more severe, enduring and treatment-resistance forms of mental illness, sometimes necessitating combinations of prescribed antipsychotics. This audit aimed to assess adherence to National Institute for Health and Care Excellence (NICE) guidelines NG28 titled “type 2 diabetes in adults: management” and summarise the metabolic parameters of those with diabetes mellitus at Broadmoor hospital.

**Method:**

This was a retrospective audit in a high secure forensic psychiatry hospital in the United Kingdom, into the management of patients with diabetes mellitus compared to guidance from NICE (NG28).

**Result:**

We report data from over 30 inpatients (out of approximately 200) at a high secure forensic psychiatry hospital with a diagnosis of type 2 diabetes mellitus across two audit cycles. This audit identified improved adherence to national guidance regarding six monthly monitoring of HbA1c but with less than 50% of such patients having an HbA1c at or below the recommended target. This is in addition to high rates of other metabolic disorders such as obesity, dyslipidaemia and hypertension and a mean QRISK3 score that was markedly higher than a healthy person with the same age, sex, and ethnicity. There was a prevalence of background diabetic retinopathy of 8%, diabetic nephropathy of 5.4%, no recorded cases of diabetic neuropathy and a macrovascular disease prevalence of 5.4%. There were no new diagnoses of microvascular or macrovascular disease between audit cycles. One of the key changes between audit cycles was the recruitment of a dietician to the hospital. By the time of undertaking the second audit cycle, 23 patients had documented evidence of having been offered a referral to the dietician.

**Conclusion:**

This audit highlighted the marked cardiovascular risk in patients with type 2 diabetes mellitus at a high secure forensic psychiatry hospital. This includes suboptimal control of blood pressure, lipid profiles and HbA1c that increases the risk of premature mortality in these patients with severe mental illness. Wider, cultural changes in practice need to be implemented to improve the metabolic health of patients in the long-term inpatient setting of Broadmoor Hospital. This includes prescribers avoiding the most diabetogenic antipsychotics where possible, increasing the provision of sugar-free options at the on-site shop, examining the proportion of carbohydrate-rich foodstuffs in the shop and understanding the characteristics of its heaviest purchasers, and continued coordination between primary care and ward teams to support patients in making sustained changes to improve their metabolic health.

